# Design and Realization of Polymeric Waveguide/Microring Structures for Telecommunication Domain

**DOI:** 10.3390/mi14051068

**Published:** 2023-05-18

**Authors:** Thuy Linh La, Binh Nguyen Bui, Thi Thanh Ngan Nguyen, Thi Lien Pham, Quoc Tien Tran, Quang Cong Tong, Aliaksandr Mikulich, Thanh Phuong Nguyen, Thi Thu Thuy Nguyen, Ngoc Diep Lai

**Affiliations:** 1 Institute of Materials Science, Vietnam Academy of Science and Technology, 18 Hoang Quoc Viet, Cau Giay, Hanoi 100000, Vietnam; 2 University of Science and Technology of Hanoi, Vietnam Academy of Science and Technology, 18 Hoang Quoc Viet, Cau Giay, Hanoi 100000, Vietnam; 3 B.I. Stepanov Institute of Physics of the National Academy of Sciences of Belarus, 68 Nezavisimosty Av., 220072 Minsk, Belarus; 4 School of Engineering Physics, Hanoi University of Science and Technology, No. 1 Dai Co Viet, Hai Ba Trung, Hanoi 100000, Vietnam; 5 Institue of Tropical Medicine, Viet Nam-Russia Tropical Center, Nguyen Van Huyen Street, Nghia Do, Cau Giay, Hanoi 100000, Vietnam; 6 LuMIn, ENS Paris-Saclay, CentraleSupélec, CNRS, Université Paris-Saclay, 91190 Gif-sur-Yvette, France

**Keywords:** waveguide, direct laser writing, FDTD, microring, optical integration

## Abstract

Polymer-based micro-optical components are very important for applications in optical communication. In this study, we theoretically investigated the coupling of polymeric waveguide and microring structures and experimentally demonstrated an efficient fabrication method to realize these structures on demand. First, the structures were designed and simulated using the FDTD method. The optical mode and loss in the coupling structures were calculated, thereby giving the optimal distance for optical mode coupling between two rib waveguide structures or for optical mode coupling in a microring resonance structure. Simulations results then guided us in the fabrication of the desired ring resonance microstructures using a robust and flexible direct laser writing technique. The entire optical system was thus designed and manufactured on a flat base plate so that it could be easily integrated in optical circuits.

## 1. Introduction

Micro-optical components, including polymer-based micro-optical components, have a large range of applications, especially in the fields of integrated optics, optical communications, optical sensors, etc. [1]. In optical communication, the bandwidth of an information transmission system is limited by the latency and response rates caused by the optical components that the system uses. Since the early 2000s, special attention has been paid to the integration of optical components and semiconductor chips to create optical integrated circuits to improve system performance. Optical integrated circuits are made using basic components, including waveguide structures, mirrors, lenses, resonant microstructures, optical filters, etc. To ensure that the signal transmission has minimal loss and that the photoconductive signal field operates in a single mode, it is important to have a fabrication technique that allows devices with arbitrary and controllable shapes and with sub-μm features to be realized. In addition, these devices should be easily integrated into optical circuits for practical uses [1].

Additionally, resonant microstructures, such as microspheres, microdiscs, and microrings, have also been intensively investigated in the past few years and are an interesting topic for optical integrated circuits [2,3]. In these structures, light can be efficiently guided and confined by Whispering Gallery Modes (WGMs), resulting in a high-quality Q-factor; WGMs are applied in numerous applications, such as low-loss lasers, efficient nonlinear optical effect, sensitive sensors, etc. Among these resonant microstructures, the microring resonator [4] is receiving more attention due to its numerous potential applications, such as optical filters, switches, routers, optical signal processing, dispersion compensation, single-mode lasers, and biosensors, and because of its capacity to be integrated into complicated optical circuits [5,6,7,8]. In wavelength division multiplexing applications, microring resonators can be used as compact wavelength filters or more complex blocks such as adaptive filter designs [9]. In order to couple light in and out of these resonant microstructures, an efficient way is to couple them with a standard waveguide. Integrated microring resonators and waveguides have been fabricated and tested on various materials, such as silicon, semiconductor, and polymer materials [10,11]. In particular, polymer materials have been proven to be a potential candidate to produce low-cost devices with a wide range of applications thanks to their advantages, such as availability, flexibility, and uncomplicated fabrication processes [9,12]. In addition, compared to semiconductor and inorganic materials [4,5,6,7,8,10,11], polymer materials could be, in a simple way, functionalized with nonlinear optical or fluorescent materials (organic, inorganic, or metal). The ensemble can be optically structured in the desired way to obtain a polymer-based photonic nanostructure (the host) containing active materials (the guest). This offers more applications at lower cost. In particular, active doped materials make it possible to tune the optical properties of a photonic device using thermal, electro-optic, and acousto-optic effects. Recent works show the advantages of using the SU8 photoresist for the fabrication of waveguides and resonant structures in the telecommunication domain [13,14]. However, these works lack the analysis and calculations to interpret and provide appropriate structural parameters, such as coupling length and coupling distance. Their fabrication, using the mask lithography technique, also limits the control of the coupling distance.

In this study, we theoretically investigated the resonant modes of polymeric waveguides and resonant microstructure-coupled polymeric waveguides and experimentally demonstrated an efficient and robust method to realize these polymeric resonant structures on demand. To evaluate the potential of these polymer-based microdevices, we first designed the structures and simulated their optical properties with the finite-difference time-domain (FDTD) algorithm [15,16] to find out optimal structure sizes and the gap distance for optical mode coupling between the structures. We then demonstrated the on-demand fabrication of these microstructures using the so-called direct laser writing (DLW) method [17,18,19], based on the low one-photon absorption (LOPA) effect.

## 2. Numerical Calculations of the Resonant Coupling of SU8 Planar Waveguides and Microring Resonators on Glass Substrate

In order to numerically calculate the optical properties of polymeric waveguides and coupled micro-resonator, we used a commercial three-dimensional, finite-difference time-domain (FDTD) solver (Ansys Lumerical software). We note that convergence tests were performed for all simulation parameters, such as the size of the FDTD simulation region, grid sizes, simulation time, and perfectly matched layer (PML) boundary conditions, to ensure reliability. Specifically, regarding grid sizes, an “override mesh region” with a grid equally divided at 5 nm resolution in all *x*-, *y*-, and *z*-directions was overridden on the entire micro-resonator and waveguide structures.

### 2.1. The Optical Mode in Polymer Waveguide Structures

**Figure 1:**
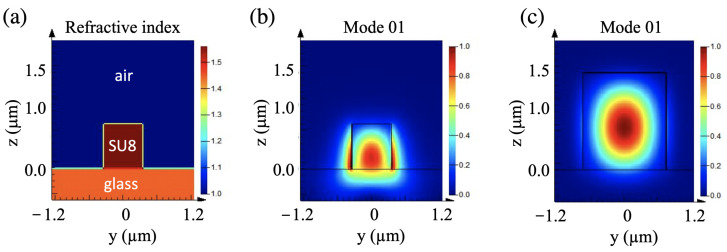
Simulation of resonant modes of polymeric waveguides using FDTD method. (**a**) Design and refractive-index profile of a polymeric waveguide having a profile of 0.7 × 0.7 μm${}^{2}$. (**b**,**c**) Field distribution of optical modes in waveguide structures with two different profiles: 0.7 × 0.7 μm${}^{2}$ (**b**) and 1.5 × 1.5 μm${}^{2}$ (**c**), respectively. The effective refractive indices of the two waveguides structures are 1.23 (**b**) and 1.44 (**c**), respectively.

A waveguide structure was designed to allow the fundamental modes to pass through with negligible loss. Compared with semiconductor materials, waveguide structures using polymer materials usually require larger sizes [20], thanks to the low-refractive-index contrast between the core and cladding of the waveguide structure. In our studies, we designed waveguide structures using a commercial photoresist, SU8, which was deposited on a standard glass substrate. The refractive indices of SU8 and glass are about 1.59 (as shown in the next section) and 1.45, respectively, in the range of the telecommunication wavelength, e.g., 1.55 μm. An example of the refractive-index profile is depicted in Figure 1a. We only considered the waveguide with a square profile of the same size in the y- and z-directions. This profile is close to that of the fabricated structures shown in the experimental section.

The fundamental waveguide modes in waveguide structures with dimensions (width and height) ranging from 0.5 μm to 3 μm were calculated. Figure 1b,c show the optical mode distribution in the polymer waveguide structures having two different widths: 0.7 μm (b) and 1.5 μm (c), respectively. In the waveguide structure of 0.7 × 0.7 μm${}^{2}$ (Figure 1b), the optical modes had optical field distributions outside of the waveguide structure, resulting in important optical loss, i.e., low optical confinement efficiency. In the 1.5 × 1.5 μm${}^{2}$ waveguide structure (Figure 1c), the optical mode was well confined within the structure. For all waveguides having sizes in the range of 0.5 μm to 3 μm, we found that they all possessed single fundamental modes. However, in small waveguide structures, the fundamental mode propagated evanescently outside the waveguide, while in those with large sizes (1.5 μm or larger), the fundamental mode was well confined within the waveguide. Therefore, the small waveguide can be usefully used as a waveguide sensor, since the evanescent field can be used to probe any change outside of the waveguide structure. However, for the application of the pure guiding effect, a polymer-based waveguide should have a larger profile, e.g., 1.5 × 1.5 μm${}^{2}$.

### 2.2. The Attenuation of Optical Signal in Polymeric Waveguide Structure

**Figure 2:**
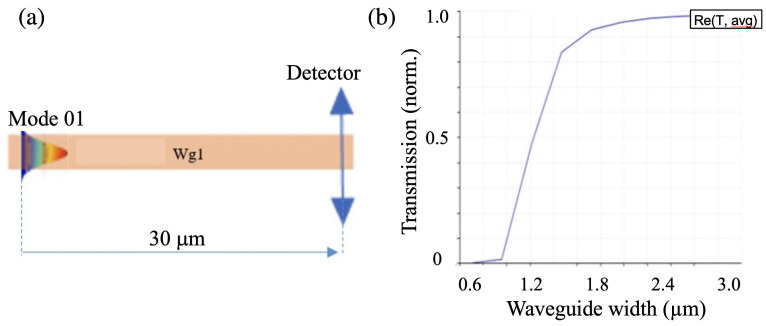
Calculation of transmission loss as a function of the waveguide width or height. (**a**) Waveguide structure simulation model. (**b**) Dependence of transmittance, *T${}_{avg}$*, on the width of the waveguide structure.

Quantitatively, we calculated the optical signal at a distance of 30 μm for different waveguides. For this calculation, we focused on the wavelength range in the telecommunication window region ($\lambda_{i}$ = 1.5–1.6 μm). The transmission signal was calculated using the following formula:

(1) $$T_{avg}=\frac{\sum_{i=1}^{n}T_{i}}{n}$$

where *n* is the number of wavelengths in the interval of 1.5–1.6 μm, $T_{i}$ is the transmission at the corresponding $\lambda_{i}$ received at a distance of 30 μm from the light source (Figure 2a), and $T_{avg}$ is the average transmittance over the wavelength range from 1.5 μm to 1.6 μm.

Figure 2b shows the simulation result of the transmittance as a function of the waveguide profile. We can see that when the width of the waveguide structure was less than 1 × 1 μm${}^{2}$, the transmittance at 30 μm was almost zero. In other words, when the waveguide is too small, most of light is leaked out due to the evanescent field, and the waveguide does not guide light as expected. When the waveguide structure width was more than 1.5 × 1.5 μm${}^{2}$, the transmittance almost reached 1, which means that the confinement of optical modes in the structure was large. However, the polymeric waveguide width should not be too large due to the possibility of having multimodes, resulting in large signal loss and consequently limited propagation distance. From the simulation results, we confirm that a good polymeric waveguide structure should have a profile in the range of 1.5 × 1.5 μm${}^{2}$ to 2.5 × 2.5 μm${}^{2}$. Therefore, in the following simulations, we chose 2 × 2 μm${}^{2}$ as the waveguide profile.

The propagation loss is also an important element of waveguide structures. It is well known that the low refractive index of polymer materials, as well as their smooth cavity/waveguide surface, induce very low loss, compared with high-refractive-index-material waveguides. Theoretically, we found a loss of the propagation power of about 0.02% for a waveguide distance of 15 μm. This corresponds to a propagation loss of about 0.58 dB/cm, which is very consistent with the result published in the literature [21]. Note that in theory, we did not consider the side-wall roughness of the waveguide or cavity. However, in practice, it is inevitable to have side-wall roughness of the fabricated structure. In the case of an SU8 polymer structure, the roughness is about a few nanometers, and it is a common situation for all fabricated waveguides. This side-wall roughness may induce a propagation loss higher than the theoretical one. However, it is well known that all SU8 polymeric waveguides can guide light for a distance of over 2 cm [22].

### 2.3. Coupling of Optical Signal in Waveguides

**Figure 3:**
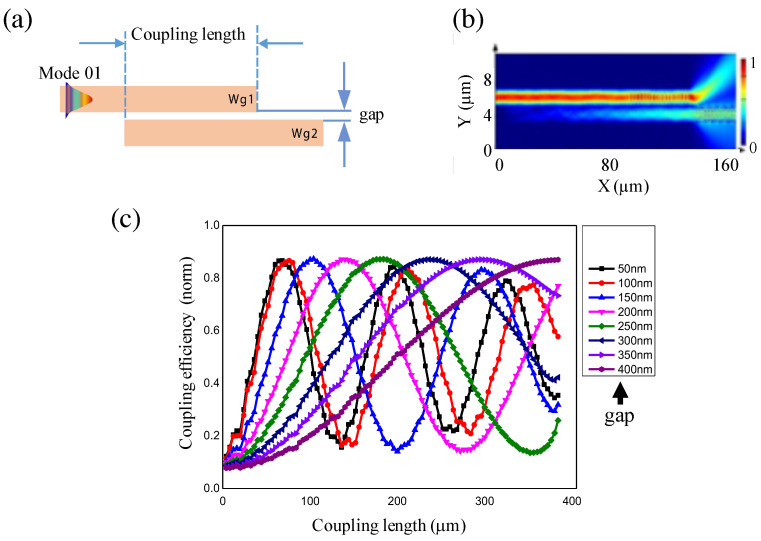
Calculation of coupling distance as a function of the gap between two waveguides. (**a**) The near-field coupling model. (**b**) Propagating optical field in the structures, corresponding to a gap of 100 nm and a coupling length of 130 μm. (**c**) Coupling efficiency versus coupling length for gaps from 50 nm to 400 nm.

In optical integrated circuits, light does not simply propagate in a single waveguide, but it is often coupled from one to another. Two optical components, such as waveguide structures or resonant microstructures, are often coupled to each other in order to manipulate light propagation or its properties, without changing the design of the waveguide structure [23]. We numerically studied the coupling of two polymeric waveguides and investigated the influence of the distance (gap) between them on coupling efficiency, as well as coupling length. Figure 3a illustrates an optical signal coupling scheme based on the near-field coupling of two linear waveguide structures made of polymer materials. Figure 3b shows the field distribution of light in the range of 1.5 μm to 1.6 μm propagating in a waveguide that was coupled to another. In this case, we assumed that two waveguides were close to each other, with a gap of 100 nm, for a distance of 130 μm. Light is not optimally coupled between two waveguides. Indeed, coupling via the near field (or evanescent field) between two waveguides strongly depends on their gaps. Each gap allows an optimal coupling distance to be obtained. We then calculated the optical coupling efficiency between the two structures using the following formula:

(2) $$\eta_{coupling}=\frac{P_{Wg2}}{P_{Wg1}}$$

where $P_{Wg1}$ and $P_{Wg2}$ are the values of the optical power of light located in the first and second waveguides, respectively.

The calculation results of near-field optical coupling efficiency are shown in Figure 3c. It is obvious that the maximum optical coupling efficiency varied with the gap width. A larger gap corresponded to a longer coupling distance. However, we also noted that the optical power varied between the two waveguides, similar to damped oscillation. It is, therefore, necessary to design two waveguides with the optimum coupling distance to avoid having light returning to the original waveguide. As seen in Figure 3c, the optimum coupling distance was in the range of hundred μm. For example, for the gap width of 100 nm, the coupling efficiency peaked at a position of 80 μm. A larger gap would increase the coupling length. This suggests that it is necessary to work with a small gap in order to fabricate compact structures. This is practically possible using our LOPA-based DLW technique, as shown in the next section.

### 2.4. Coupling between a Resonant Microring Structure and Waveguides

**Figure 4:**
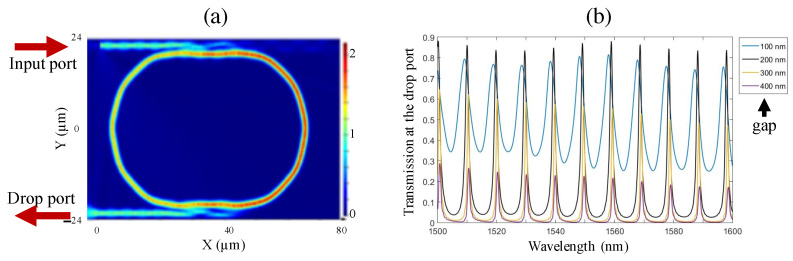
(**a**) Electrical field distribution in the micro-racetrack resonance structure at the resonance wavelength of 1591 nm. The waveguide/cavity has a cross-section of 2 × 2 μm${}^{2}$; the gap between them is 200 nm, and the coupling distance is 20 μm. (**b**) Transmission spectra at drop port of the micro-racetrack resonance structure having different gaps: 100 nm, 200 nm, 300 nm, and 400 nm, respectively.

Micro-resonators, such as microrings or microspheres, are very important optical devices that allow one to selectively filter out the desired wavelengths and amplify the field. The combination of two linear waveguides and a micro-resonator makes it possible to couple the light in and out of the micro-resonator. Such a combined system has many applications, including interferometers, lasers, optical filters, optical delays, etc. [3,24,25]. In the simulation model, we used a micro-racetrack resonance structure coupled with two rib waveguides. The waveguide structures and the micro-resonator had a cross-section of 2 × 2 μm${}^{2}$, as previously mentioned. A 1.5–1.6 μm wide-band light source was introduced into the input port. The optical signal was coupled into the micro-racetrack resonance structure and received at the output port. Only wavelengths satisfying the resonance conditions in a microring resonator structure can be enhanced to form resonant modes, while the remaining wavelengths are significantly suppressed or attenuated during propagation through the structure. Figure 4a shows an example of a coupled structure where the gap between the waveguide and the micro-resonator is 200 nm and the coupling distance is 20 μm. The electrical field distribution was plotted using a resonance mode at the 1591 nm wavelength. Similar to the case of coupling between two waveguides, efficient coupling depends on different parameters. We then fixed the coupling distance at 20 μm and changed the gap between the waveguides and the micro-resonator. Figure 4b shows the transmission spectra received at the drop port for the different gaps of 100, 200, 300, and 400 nm. We clearly see that the micro-resonator makes it possible to selectively couple some particular wavelengths, i.e., resonant modes, while in other wavelength regions, the signal is significantly attenuated. Obviously, this coupling system was not optimum, i.e., it did not allow light to be coupled in and out with maximum signal, for small and large gaps. Indeed, with a 100 nm gap, the resonant peaks were wide and have low contrast, while with a 400 nm gap, the transmitted signal at the resonant wavelength became weaker. With a 20 μm coupling length, we conclude that the optimum gap is about 200 nm, which results in high signal intensity at resonance peaks and a narrow spectrum.

We note that the Q-factor of this resonant system based on polymer material was not as high as that obtained with high-refractive-index material. Nevertheless, by using a longer resonator that was fabricated using the mask lithography technique, a high Q-factor of about 10${}^{5}$ was obtained [22]. However, the finesse, defined as the free spectral range (FSR) divided by the linewidth of the resonances, of a resonator depends on the length of the resonator, as shown by the following formula:

(3) $$FSR\approx \frac{\lambda^{2}}{n_{eff}L}$$

where λ is the wavelength, $n_{eff}$ is the effective refractive index, and *L* is the length of the resonator. This means that a long resonator results in small finesse. Therefore, we expect that the small cavity fabricated using our DLW method could provide both optimum parameters, i.e., high Q-factor and large finesse.

## 3. Fabrication of Racetrack Microring Resonators on SU8/Glass

**Figure 5:**
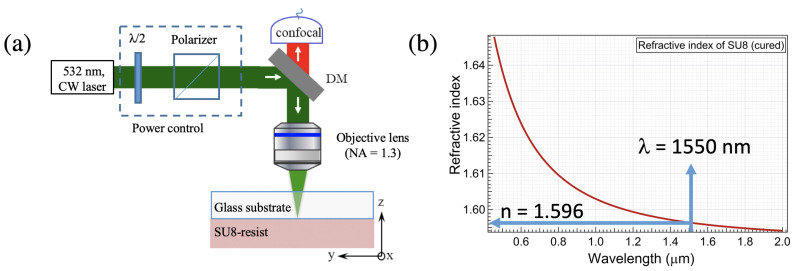
(**a**) The schematic diagram of low one-photon absorption (LOPA)-based direct laser writing (DLW) technique. (**b**) Measurement of refractive index of photopolymerized SU8 photoresist.

The waveguides and waveguide-coupled micro-resonator can be fabricated using various methods, for example, using the mask lithography technique or the imprinting technique. However, these techniques require multiple steps to obtain a waveguide or waveguide-coupled micro-resonator structures. Moreover, the structures and their coupling parameters cannot be controlled when the template is fabricated. In this work, we demonstrate the fabrication of these structures on demand, in a single step, using the DLW technique based on the LOPA effect, which is very flexible and low-cost [26]. The principle of LOPA-based DLW is shown in Figure 5a. The writing system uses a diode-pumped, solid-state, doubled-frequency Nd:YAG continuous laser with a maximum power of 300 mW. The laser wavelength is 532 nm, corresponding to the low absorption range of the commercial SU8 photoresist, i.e., the LOPA regime. The SU8 resist is deposited on a glass sample, with a standard preparation process. The thickness of the SU8 film can be controlled from 0.5 to 25 μm, depending on the desired structures. The refractive index of SU8 films is measured before and after the photopolymerization process. Figure 5b shows the refractive index versus wavelengths of a polymerized sample. The refractive index was about 1.590 in the telecommunication wavelength, which was larger than that of the glass substrate (about 1.45 in the same wavelength range). The measured refractive index is consistent with the result published in [27] for the same material and wavelength. The laser beam is focused by an objective lens with high numerical aperture (NA = 1.3, oil immersion) to a diffraction-limited focusing spot of about 250 nm. We note that the real fabricated feature can be smaller than the diffraction limit, since the polymerized structure depends not only on the light-focusing spot but also on the nonlinear polymerization mechanism [26]. By driving the focusing beam in the polymer, point by point and layer by layer, using a high-resolution, three-dimensional piezo-electrical translation (PZT) system, any desired structure can be created.

**Figure 6:**
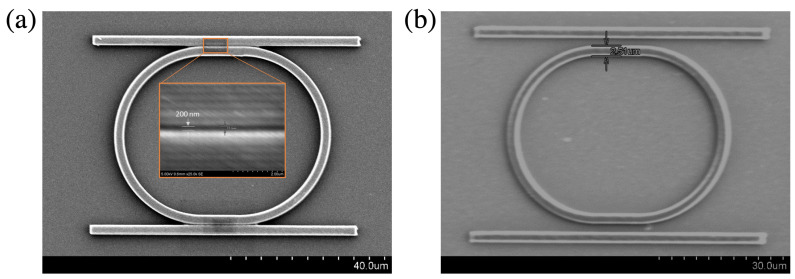
SEM images of microring resonators coupled to rib waveguides with different gaps. (**a**) Gap = 200 nm. The SEM image at the center shows a magnification of the gap. (**b**) Gap = 2000 nm. The coupled structures were fabricated using the LOPA-based DLW technique on the SU8 photoresist.

Figure 6 shows the scanning electron microscope (SEM) images of two examples of microring resonator structures with radius *r* = 20 μm; a coupling length of 20 μm; and two gaps, 200 nm and 2000 nm, respectively. The same fabrication parameters were used for the fabrication of these samples (laser power *P* = 3 mW and average scanning speed of 5 μm/s; the thickness of the SU8 film was 2 μm). This example demonstrates that LOPA-DLW can produce any structure calculated in the simulation section with high accuracy.

**Figure 7:**
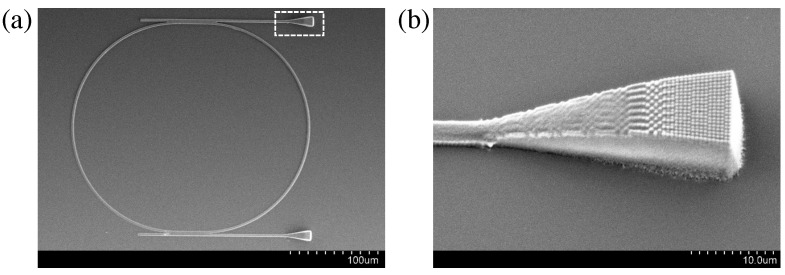
SEM image of a microring resonator coupled to rib waveguides. The waveguide input/output is added using grating/taper coupling. (**a**) Full image. (**b**) Magnification of one input or output, as indicated by the white frame in (**a**). The coupled structures were fabricated using the LOPA-based DLW technique on the SU8 photoresist line by line with a gap of 500 nm.

Moreover, even if we do not show any theoretical calculation, the coupling light from an external source to the waveguide is also a crucial step. We, therefore, demonstrated that LOPA-DLW can also fabricate a waveguide structure, including grating/taper coupling, as shown in Figure 7. This grating/taper coupling plays the role of a focusing lens, allowing a large external light beam to be focused into the waveguide, whose diameter is in the range of micrometers. For this fabrication, a thick SU8 film (about 10 μm) was used to obtain a thick grating/taper as shown in Figure 7b. The “diffraction grating” was fabricated line by line with a gap of 500 nm. This is just a demonstration of the flexibility of the DLW technique. The optimum grating structure could be calculated and re-fabricated in the near future for an experimental characterization experiment. We note that there is no difficulty in fabricating optimum gratings having a period in the range of micrometers, since the target wavelength is about 1.55 μm.

**Figure 8:**
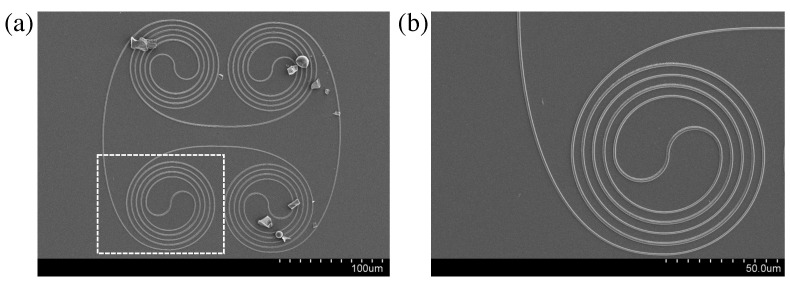
SEM image of a complex microring resonator showing the powerful possibility of using the LOPA-based DLW fabrication technique. (**a**) Full image. (**b**) Magnification of one part as indicated by the white frame in (**a**). In (**a**), some dirty particles exist on the microring resonator due to the sample cutting process.

Finally, in order to overcome the limited scanning range of PZT, which is typically 100 × 100 × 100 μm${}^{3}$, and to obtain a long resonant microring system, we show, in Figure 8, a possible polymer-based micro-resonator system, which was also fabricated using the LOPA-based DLW method. This could make photonic systems very compact, and it possesses a short FSR, since the cavity length is long. Again, this confirms that LOPA-DLW is very robust for the fabrication of such integrated photonic circuits.

It is worth to note that since the waveguide resonant structure fabricated using DLW is quite small, typically 10–100 μm in size, we need a special characterization system to obtain their experimental spectra or to measure the propagation loss. It very difficult for us to do so at this moment, but we hope to report it in the very near future.

## 4. Conclusions

In conclusion, with the design of a waveguide structure based on a low-refractive-index material, SU8, coated on a glass substrate, we calculated the optical coupling efficiency using the near-field effect with the FDTD method. Optimum optical coupling efficiency was determined as a function of the waveguide cross-section, as well as the coupling length and coupling gap. This performance has important implications for the design and fabrication of optical integrated circuit structures. Different waveguides and waveguide-coupled micro-resonators were fabricated on demand using the direct laser writing technique based on the LOPA mechanism. This work shows an important step towards the development of optical integrated circuits in the telecommunication domain.

## Abbreviations

The following abbreviations are used in this manuscript:

DLW	Direct laser writing
SEM	Scanning electron microscope
NA	Numerical Aperture
PZT	Piezo-electrical ceramic
FSR	Free spectral range
FDTD	Finite-difference time-domain
WGM	Whispering Gallery Modes
